# Association between the *LRP5* rs556442 gene polymorphism and the risks of NAFLD and CHD in a Chinese Han population

**DOI:** 10.1186/s12876-022-02385-9

**Published:** 2022-06-22

**Authors:** Dongli Han, Haiying Zhang, Shousheng Liu, Likun Zhuang, Zhenzhen Zhao, Hongguang Ding, Yongning Xin

**Affiliations:** 1 Department of Infectious Disease, Qingdao Municipal Hospital, 1 Jiaozhou Road, Qingdao, 266011 Shandong Province China; 2 Health Management Center, Qingdao Central Hospital, Qingdao, China; 3 Clinical Research Center, Qingdao Municipal Hospital, Qingdao, China; 4 Second Department of General Surgery, Qingdao Municipal Hospital, 1 Jiaozhou Road, Qingdao, 266011 Shandong Province China; 5 Digestive Disease Key Laboratory of Qingdao, Qingdao, China; 6 Department of Gastroenterology, Zhumadian Central Hospital, Zhumadian, China

**Keywords:** Coronary atherosclerotic heart disease, LRP5, Non-alcoholic fatty liver disease, Polymorphism

## Abstract

**Background:**

Multiple studies have demonstrated the involvement of low-density lipoprotein receptor-related protein 5 (LRP5) in metabolism-related diseases. This study explored the relationship between the *LRP5* rs556442 gene polymorphism and the risks of non-alcoholic fatty liver disease (NAFLD) and coronary heart disease (CHD) in a Chinese Han population.

**Methods:**

This retrospective case–control study included 247 patients with NAFLD, 200 patients with CHD, 118 patients with both NAFLD and CHD, and 339 healthy controls from June 2018 to June 2019 at Qingdao Municipal Hospital. Basic information and clinical characteristics were collected for all subjects. The genotype and allele frequency of *LRP5* rs556442 were determined.

**Results:**

The genotype distributions of *LRP5* rs556442 differed significantly between the CHD and NAFLD + CHD groups (*P* < 0.05). The *LRP5* rs556442 GG genotype markedly promoted the risk of NAFLD in CHD patients [odds ratio (OR) = 2.857, 95% confidence interval (CI): 1.196–6.824, *P* = 0.018). After adjustment for sex, age, and body mass index (BMI), this association remained significant (OR = 3.252, 95% CI: 1.306–8.102, *P* = 0.011). In addition, the *LRP5* rs556442 AA + AG genotype was associated with an increased BMI in obese NAFLD patients (OR = 1.526, 95% CI: 1.004–2.319, *P* = 0.048). However, after adjustment for sex and age, this association was no longer significant (OR = 1.504, 95% CI: 0.991–2.282, *P* = 0.055).

**Conclusions:**

This study found that the *LRP5* rs556442 GG genotype increased the risk of NAFLD in CHD patients and AA + AG genotype may be associated with an increased BMI in obese NAFLD patients among a Chinese Han population.

*Trial registration* ChiCTR, ChiCTR1800015426. Registered 28 March 2018—Retrospectively registered, http://www.chictr.org.cn/showproj.aspx?proj=26239.

## Background

NAFLD is the most prevalent chronic liver disease and may progress to NAFLD-associated cirrhosis [1]. NAFLD cases are usually classified as lean NAFLD or obese NAFLD based on the BMI value of 25 kg/m^2^ [2]. The recent studies suggested that the prevalence of NAFLD in Asia is about 29.62% [3, 4]. In addition, an increasing number of adolescents are being diagnosed with NAFLD, with an estimated prevalence rates ranging from 3–18% [3, 5]. A sedentary lifestyle, poor diet, and metabolism-related diseases are the main reasons for the elevated morbidity of NAFLD, and NAFLD is currently the main condition requiring liver transplantation [6, 7]. At present, no targeted drug is available for the treatment of NAFLD, and the primary means to improve the symptoms of NAFLD are weight loss and improvement of insulin resistance through lifestyle, medication, or endoscopic/surgical interventions [1, 8, 9].

CHD is the main cause of myocardial ischemia, which is closely correlated with the occurrence of major adverse cardiovascular events [10]. CHD has become the main cause of death in patients with NAFLD, and a prospective cohort study found that NAFLD is tightly related to CHD and an independent risk factor for CHD [11, 12]. An imaging study also detected a strong relationship between CHD and NAFLD [13]. In a prospective cohort study by Wong et al., the prevalence of NAFLD among patients with CHD was 58.2% [14]. Arslan et al. also demonstrated a correlation between NAFLD and CHD [15]. NAFLD and CHD are interrelated through complex pathophysiological mechanisms [16]. As genetic factors have been recognized to play important roles in precision medicine, some common risk genes have been reported for these two diseases, including *TRIB1* rs17321515, *ADIPOQ* rs266729, *PNPLA3* rs738409, and *LEPR* rs1137100 [17–23].

LRP5 is located on chromosome 11, and the rs556442 variant is present in the 15th exon of the *LRP5* gene [24]. LRP5 is expressed in various tissues, including the liver and pancreatic β-cells [25, 26], participates in the process of adipogenesis by down-regulating adipogenic transcription factors, and also regulates the process of glucose-induced insulin secretion and cholesterol metabolism [27]. Montazeri-Najafabady et al. reported that the *LRP5* rs556442 polymorphism increases the risk of insulin resistance in Iranian adolescents and emphasized that the A allele played a key role in the increase in total cholesterol (TC) levels in the study population [28, 29]. In 2019, Adabi et al. showed that the *LRP5* rs556442 variant could affect the basal metabolic rate [30]. It is well known that hypercholesterolemia, insulin resistance, and obesity all contribute to NAFLD and play important roles in the occurrence and development of CHD. In view of the known functional characteristics of *LRP5* rs556442, it is reasonable to presume that *LRP5* rs556442 may influence the risks of NAFLD and CHD, but no available study has proven this. This study aimed to explore the correlation between *LRP5* rs556442 and the risks of NAFLD and CHD in a Chinese Han population, and to investigate the effect of the *LRP5* rs556442 A allele specifically on metabolism-related parameters.

## Methods

### Research subjects

In this retrospective case–control study, all subjects were treated from June 2018 to June 2019 in Qingdao Municipal Hospital. NAFLD was diagnosed according to the Guidelines of Prevention and Treatment for Nonalcoholic Fatty Liver Disease: a 2018 update [31]. Patients with NAFLD were recruited from the department of Infectious Disease and Gastroenterology in Qingdao Municipal Hospital. NAFLD was diagnosed based on basic features such as an enhanced anterior field echo (bright liver), attenuation of distant field echo, and an unclear intrahepatic duct structure observed on abdominal ultrasound. Patients with alcoholic liver disease, viral liver disease, autoimmune liver disease, drug-induced liver disease, and other related liver diseases were excluded from this study. CHD was diagnosed based on the 2015 Chinese Society of Cardiology (CSC) guidelines for the diagnosis and management of patients with ST-segment elevation myocardial infarction, and patients were diagnosed with CHD when coronary angiography showed stenosis more than 50% in any of the main coronary arteries. Patients with both NAFLD and CHD (NAFLD + CHD) met both the criteria for diagnosis of both NAFLD and CHD. Patients with CHD or NAFLD + CHD were recruited from the department of Cardiology in Qingdao Municipal Hospital. Healthy controls were recruited from the Health Management Center in Qingdao Municipal Hospital. NAFLD was assessed by a hepatologist, and CHD was assessed by a cardiologist, all of whom were blinded to the study aims and patient details. A total of 339 healthy controls, 247 patients with NAFLD, 200 with CHD, and 118 with both NAFLD and CHD were included. All participants were of Chinese Han ethnicity. This study was approved by the ethics committee of Qingdao Municipal Hospital and performed in accordance with the Declaration of Helsinki and its amendments [32].

### Biochemical analyses

Blood samples were collected from participants after 12 h of overnight fasting. The levels of biochemical parameters such as high-density lipoprotein (HDL), aspartate aminotransferase (AST), fasting plasma glucose (FPG), TC, alanine aminotransferase (ALT), triglycerides (TG), low-density lipoprotein (LDL), alkaline phosphatase (ALP), total bilirubin (TB), and γ-glutamyl transpeptidase (GGT) were measured in the laboratory medicine department. All basic information for the participants was retrieved from a questionnaire.

### Genomic DNA extraction and genotyping

For the genotyping, genomic DNA of blood samples was extracted using a commercial kit (TIANGEN, Beijing, China). Polymerase chain reaction (PCR) amplification for *LRP5* rs556442 was performed used the primers S: 5′-ACGTTGGATGTACTGAAATCACTGTCCCTC-3′; AS: 5′-ACGTTGGATGAACAAGCACTTCGGTCATCC-3′, and the procedure previous described by Chen et al. [33]. After PCR amplification, the PCR products were treated with alkaline phosphatase before single-base extension reaction and resin purification. Matrix-assisted laser desorption ionization time-of-flight (MALDI-TOF) mass spectrometry was applied to analyze the spectral chip after sampling, and the MassARRAY TYPER 4.0 software was used to analyzed the raw data.

### Statistical analysis

Statistical analyses were performed using SPSS 23.0 software (SPSS, Inc., USA). Hardy–Weinberg equilibrium was applied to confirm balance of genotype frequencies among the groups. Genotype distribution, allele frequency, and sex were compared among groups using the χ^2^ test. Data for variables that followed a normal distribution are expressed as mean ± standard deviation (SD), and data for variables that did not are expressed as quartiles. If the data were normally distributed and F test showed equal variance, differences in continuous variables between two groups were identified using t tests. Otherwise, the Wilcoxon test was used. Also, if the data were normally distributed and F test showed equal variance, the differences in continuous variables in two of the four groups were identified using Student–Newman–Keuls test. Otherwise, the Kruskal–Wallis test was used. OR and 95% CI values were calculated by the binary logistic regression model. *P* < 0.05 was considered as the statistic difference.

## Results

### Characteristics of subjects in this study

Data for 904 individuals were analyzed in this retrospective study, including 247 patients with NAFLD (139 males and 108 females), 200 patients with CHD (127 males and 73 females), 118 patients with both NAFLD and CHD (81 males and 37 females), and 339 healthy controls (188 males and 151 females). The age and gender of NAFLD patients did not differ from those of the control individuals. The levels of FPG, TC, ALT, AST, ALT/AST, GGT, and ALP as well as BMI in NAFLD patients were elevated compared with those of the control group, while the levels of TB and HDL in NAFLD patients were markedly decreased compared with those of controls (all *P* < 0.05). The levels of ALT, GGT, FPG, AST, and ALP in addition to the age and BMI of CHD patients were elevated compared with those of the controls, while the levels of LDL and HDL were lower in the CHD patients than in the controls (all *P* < 0.05). The proportion of male patients in the NAFLD + CHD patients was higher than that in the controls (*P* < 0.05). Additionally, the levels of FPG, ALT, GGT, and ALP as well as the age and BMI of the NAFLD + CHD patients were higher than that in controls, while the levels of HDL and LDL in NAFLD + CHD patients were inferior to that in controls (all *P* < 0.05). The proportion of male patients in NAFLD group was higher than that in the NAFLD + CHD group (*P* < 0.05). Age and FPG concentration were higher in NAFLD + CHD patients than in NAFLD patients, whereas BMI, the ALT/AST ratio, and the concentrations of TC, HDL, and LDL were lower in the NAFLD + CHD patients than in the NAFLD patients (all *P* < 0.05). No significant differences in gender and age were observed between the NAFLD + CHD patients and the CHD patients (both *P* > 0.05), and only the TG level in the NAFLD + CHD patients was elevated compared with that in CHD patients (*P* < 0.05) (Table 1).

**Table 1:** Clinical characteristics in the NAFLD, CHD, NAFLD + CHD, and control groups

	NAFLD	CHD	NAFLD + CHD	Controls	*P*_1_	*P*_2_	*P*_3_	*P*_4_	*P*_5_
Sex (M/F)	247 (139/108)	200 (127/73)	118 (81/37)	339 (188/151)	0.844	0.067	0.012	0.024	0.352
Age, years	48.38 ± 11.98	67.12 ± 11.52	61.59 ± 8.06	46.48 ± 12.04	1.000	< 0.001	< 0.001	< 0.001	0.057
BMI, kg/m^2^	26.50 ± 2.87	24.51 ± 3.19	25.10 ± 2.45	23.46 ± 3.39	< 0.001	0.002	< 0.001	0.002	0.503
FPG, mmol/L	4.76 (4.41–5.28)	5.22 (4.55–6.76)	5.41 (4.72–6.48)	4.59 (4.17–5.05)	0.003	< 0.001	< 0.001	< 0.001	1.000
TC, mmol/L	5.31 ± 1.16	4.64 ± 1.31	4.41 ± 1.34	4.30 ± 1.94	< 0.001	1.000	0.397	< 0.001	1.000
TG, mmol/L	1.53 (1.13–2.14)	1.38 (0.97–1.85)	1.46 (1.00–2.27)	1.39 (0.91–3.87)	0.352	0.092	0.816	0.740	0.042
HDL, mmol/L	1.20 (1.05–1.32)	1.02 (0.86–1.16)	1.02 (0.85–1.16)	1.36 (1.14–1.55)	< 0.001	< 0.001	< 0.001	< 0.001	1.000
LDL, mmol/L	3.45 ± 1.66	2.79 ± 1.09	2.71 ± 0.90	3.22 ± 0.71	1.000	< 0.001	< 0.001	< 0.001	1.000
ALT, U/L	24.74 (17.40–38.81)	21.71 (14.73–31.73)	22.42 (15.38–33.37)	18.14 (13.39–24.68)	< 0.001	0.003	0.006	0.221	1.000
AST, U/L	22.66 (18.67–28.16)	22.65 (17.15–34.81)	22.42 (16.40–32.69)	20.33 (17.22–24.33)	0.001	0.002	0.050	1.000	1.000
ALT/AST	1.13 (0.88–1.45)	0.92 (0.64–1.23)	0.95 (0.68–1.23)	0.91 (0.72–1.14)	< 0.001	1.000	1.000	< 0.001	1.000
GGT, U/L	46.25 ± 79.71	33.33 ± 28.60	47.15 ± 77.70	2.28 ± 29.51	< 0.001	< 0.001	< 0.001	0.333	1.000
ALP, U/L	77.29 (61.80–93.86)	83.51 (65.80–106.56)	82.20 (71.10–98.63)	65.54 (53.09–80.34)	< 0.001	< 0.001	< 0.001	0.063	1.000
TB, μmol/L	13.29 ± 5.13	14.36 ± 6.83	14.98 ± 7.95	14.61 ± 5.66	0.007	1.000	1.000	0.798	1.000

*NAFLD* non-alcoholic fatty liver disease, *CHD* coronary heart disease, *BMI* body mass index, *FPG* fasting blood glucose, *TC* total cholesterol, *TG* triglycerides, *HDL* high-density lipoprotein, *LDL* low-density lipoprotein, *ALT* alanine aminotransferase, *AST* aspartate aminotransferase, *GGT*, γ-glutamyl transpeptidase, *ALP* alkaline phosphatase, *TB* total bilirubin

*P*_*1*_: NAFLD patients vs. healthy controls; *P*_*2*_: CHD patients vs. healthy controls; *P*_*3*_: NAFLD + CHD patients vs. healthy controls; *P*_*4*_: NAFLD patients vs. NAFLD + CHD patients; *P*_*5*_: CHD patients vs. NAFLD + CHD patients

### Distribution of LRP5 rs556442 genotype and allele frequency

The genotype distributions of *LRP5* rs556442 among the four groups conformed to Hardy–Weinberg equilibrium, no significant differences of genotype distributions of *LRP5* rs556442 were found in NAFLD group, CHD group, NAFLD + CHD group, and Control group (all *P* > 0.05). In the CHD group, the genotype distribution of *LRP5* rs556442 was significantly different from that in the NAFLD + CHD group (*P* < 0.05), but the difference of allele frequency distribution of *LRP5* rs556442 was not significant between CHD and NAFLD + CHD groups (*P* > 0.05). No significant differences in allele frequency and genotype distribution of *LRP5* rs556442 were found between NAFLD patients and controls, CHD patients and controls, NAFLD + CHD patients and healthy controls, NAFLD patients and NAFLD + CHD patients, or lean NAFLD patients and obese NAFLD patients (Table 2). Binary logistic regression analysis suggested a significant association between the *LRP5* rs556442 GG genotype and the risk of NAFLD in CHD patients (OR = 2.857, 95% CI: 1.196–6.824, *P* = 0.018), and this association remained significant after adjustment for age, sex, and BMI (OR = 3.252, 95% CI: 1.306–8.102, *P* = 0.011; Table 3).

**Table 2:** Distributions of *LRP5* rs556442 genotypes and allele frequencies in the four groups^a^

	NAFLD			CHD	NAFLD + CHD	Control	*P*_1_	*P*_2_	*P*_3_	*P*_4_	*P*_5_	*P*_6_
	All NAFLD	Lean NAFLD	Obese NAFLD									
Genotype frequency, n (%)												
AA	126 (51.01)	37 (50.00)	89 (51.45)	102 (51.00)	59 (50.00)	174 (51.33)	0.986	0.260	0.436	0.417	0.043	0.793
AG	102 (41.30)	30 (40.54)	72 (41.62)	89 (44.50)	45 (38.14)	138 (40.71)						
GG	19 (7.69)	7 (9.46)	12 (6.93)	9 (4.50)	14 (11.86)	27 (7.96)						
Allele frequency, n (%)												
A	354 (71.66)	104 (70.27)	250 (72.25)	150 (71.43)	163 (69.07)	486 (71.68)	0.994	0.578	0.446	0.471	0.258	0.654
G	140 (28.34)	44 (29.73)	96 (27.74)	60 (28.57)	73 (30.93)	192 (28.32)						

*NAFLD* non-alcoholic fatty liver disease, *CHD* coronary heart disease

*P*_*1*_: NAFLD patients vs. healthy controls; *P*_*2*_: CHD patients vs. healthy controls; *P*_*3*_: NAFLD + CHD patients vs. healthy controls; *P*_*4*_: NAFLD patients vs. NAFLD + CHD patients; *P*_*5*_: CHD patients vs. NAFLD + CHD patients; *P*_*6*_*:* lean NAFLD patients vs. obese NAFLD patients

^a^Data were compared by chi-square test

**Table 3:** Correlations of genotypes with risks of NAFLD and CHD in the study groups

	OR (95% CI)	*P*_1_	OR (95% CI)	*P*_2_	OR (95% CI)	*P*_3_	OR (95% CI)	*P*_4_	OR (95% CI)	*P*_5_	OR (95% CI)	*P*_6_
AA + AG	1	0.904	1	0.124	1	0.205	1	0.197	1	0.018	1	0.497
GG	0.963 (0.523–1.774)		0.545 (0.251–1.183)		1.556 (0.786–3.078)		1.615 (0.780–3.346)		2.857 (1.196–6.824)		0.713 (0.268–1.891)	
Adjusted^a^												
AA + AG	1	0.476	1	0.752	1	0.096	1	0.648	1	0.011	1	0.650
GG	1.285 (0.645–2.560)		0.840 (0.286–2.470)		2.147 (0.874–5.273)		1.236 (0.498–3.066)		3.252 (1.306–8.102)		0.789 (0.284–2.192)	

*OR* odds ratio, *CI* confidence interval

*P*_*1*_: NAFLD patients vs. healthy controls; *P*_*2*_: CHD patients vs. healthy controls; *P*_*3*_: NAFLD + CHD patients vs. healthy controls; *P*_*4*_: NAFLD patients vs. NAFLD + CHD patients; *P*_*5*_: CHD patients vs. NAFLD + CHD patients; *P*_*6*_*:* lean NAFLD patients vs. obese NAFLD patients

^a^Binary logistic regression model was adjusted for age, gender, and BMI

### Association between LRP5 rs556442 A allele and clinical parameters in each group

Among all study participants and among NAFLD patients, *LRP5* rs556442 A allele carriers possessed an elevated BMI compared to non-carriers (both *P* < 0.05). In addition, no significant differences in other indicators were observed between carriers and non-carriers in these groups. *LRP5* rs556442 A allele carriers had an elevated AST level compared to non-carriers among NAFLD + CHD patients (*P* < 0.05), but the differences in other indicators were not significant between carriers and non-carriers in this group (Table 4). In addition, no significant differences in clinical parameters were observed between the carriers and non-carriers of A allele among CHD patients or controls. NAFLD cases were divided into lean NAFLD and obese NAFLD according to BMI. Binary logistic regression analysis showed that the *LRP5* rs556442 AA + AG genotype was associated with the increased BMI in obese NAFLD patients (OR = 1.526, 95% CI: 1.004–2.319, *P* = 0.048). However, this association was not significant after adjustment for sex and age (OR = 1.504, 95% CI: 0.991–2.282, *P* = 0.055; Table 5).

**Table 4:** Clinical characteristics of *LRP5* rs556442 A allele carriers and non-carriers in the study population

	All study participants			NAFLD patients			NAFLD + CHD patients		
	Carriers	Non-carriers	*P*	Carriers	Non-carriers	*P*	Carriers	Non-carriers	*P*
Sex (M/F)	835 (501/334)	69 (34/35)	0.082	228 (130/98)	19 (9/10)	0.415	104 (71/33)	14 (10/4)	0.811
Age, years	53.61 ± 14.37	52.71 ± 14.37	0.618	48.31 ± 11.90	49.26 ± 13.15	0.739	61.5 ± 8.07	62.29 ± 8.22	0.734
BMI, kg/m^2^	24.84 ± 3.23	23.41 ± 4.17	0.001	26.59 ± 2.93	25.44 ± 1.88	0.022	25.21 ± 2.45	24.25 ± 2.34	0.168
FPG, mmol/L	4.84 (4.37–5.56)	4.74 (4.39–5.20)	0.261	4.77 (4.40–5.28)	4.74 (4.46–5.28)	0.781	5.43 (4.77–6.49)	4.95 (4.33–6.39)	0.310
TC, mmol/L	4.46 ± 1.64	4.68 ± 1.60	0.263	5.32 ± 1.17	5.16 ± 1.15	0.551	4.41 ± 1.36	4.37 ± 1.22	0.954
TG, mmol/L	1.45 (1.01–2.23)	1.25 (0.87–1.94)	0.110	1.57 (1.15–2.19)	1.22 (0.99–1.90)	0.530	1.49 (0.97–2.32)	1.38 (1.18–2.18)	0.97
HDL, mmol/L	1.17 (1.00–1.38)	1.20 (1.01–1.48)	0.357	1.20 (1.05–1.32)	1.20 (1.08–1.45)	0.355	1.02 (0.87–1.16)	0.96 (0.83–1.19)	0.638
LDL, mmol/L	3.11 ± 0.99	3.12 ± 1.19	0.954	3.48 ± 1.71	3.06 ± 0.70	0.292	2.71 ± 0.91	2.73 ± 0.83	0.916
ALT, U/L	20.98 (15.00–31.16)	17.93 (12.92–28.62)	0.051	24.83 (17.63–39.02)	22.67 (13.85–37.13)	0.460	22.71 (15.62–33.51)	18.69 (10.93–29.73)	0.201
AST, U/L	21.47 (17.95–27.67)	20.71 (16.02–25.62)	0.092	22.87 (18.67–28.29)	21.55 (18.24–28.09)	0.729	23.30 (16.94–34.48)	18.63 (14.41–25.24)	0.001
ALT/AST	0.97 (0.75–1.27)	0.92 (0.68–1.10)	0.184	1.15 (0.88–1.46)	1.07 (0.81–1.24)	0.331	0.94 (0.68–1.24)	1.01 (0.69–1.25)	0.693
GGT, U/L	27.10 ± 21.15	37.88 ± 57.47	0.122	47.66 ± 82.71	29.30 ± 14.66	0.336	48.31 ± 81.49	38.49 ± 40.38	0.659
ALP, U/L	74.80 (59.82–91.52)	74.33 (59.55–97.60)	0.916	77.38 (62.55–93.59)	63.86 (51.98–105.33)	0.326	82.26 (69.75–99.42)	81.44 (73.38–98.63)	0.884
TB, μmol/L	14.69 ± 6.86	14.21 ± 6.11	0.53	13.31 ± 5.14	13.04 ± 5.15	0.828	15.14 ± 8.27	13.77 ± 5.03	0.547

*NAFLD* non-alcoholic fatty liver disease, *CHD* coronary heart disease, *BMI* body mass index, *FPG* fasting blood glucose, *TC* total cholesterol, *TG* triglycerides, *HDL* high-density lipoprotein, *LDL* low-density lipoprotein, *ALT* alanine aminotransferase, *AST* aspartate aminotransferase, *GGT*, γ-glutamyl transpeptidase, *ALP* alkaline phosphatase; TB, total bilirubin

**Table 5:** Associations between AA + AG genotype and BMI in all study participants and in NAFLD patients by binary logistic regression analysis

	All study participants		NAFLD patients					
			All NAFLD		Lean NAFLD		Obese NAFLD	
	OR (95% CI)	*P*	OR (95% CI)	*P*	OR (95% CI)	*P*	OR (95% CI)	*P*
Model 1		0.001		0.096		0.754		0.048
GG	1		1		1		1	
AA + AG	1.130 (1.053–1.213)		1.162 (0.974–1.387)		0.899 (0.467–1.750)		1.526 (1.004–2.319)	
Model 2		0.002		0.133		0.721		0.055
GG	1		1		1		1	
AA + AG	1.120 (1.043–1.203)		1.151 (0.958–1.382)		0.886 (0.455–1.723)		1.504 (0.991–2.282)	

Model 1: uncorrected; Model 2: adjusted for sex and age

*NAFLD* non-alcoholic fatty liver disease, *OR* odds ratio, *CI* confidence interval

## Discussion

Accumulated studies have proven that the *LRP5* rs556442 polymorphism tightly associated with the risk of multiple metabolic related diseases, but the correlation between *LRP5* rs556442 and the risks of NAFLD and CHD in a Chinese Han population remain unclear. This study investigated the relationship between the *LRP5* rs556442 gene polymorphism and the risks of non-alcoholic fatty liver disease (NAFLD) and coronary heart disease (CHD) in a Chinese Han population. The results showed that *LRP5* rs556442 GG genotype markedly promoted the risk of NAFLD in CHD patients, and the AA + AG genotype may be associated with an increased BMI in obese NAFLD patients among a Chinese Han population.

Previous studies have suggested that NAFLD and CHD are interrelated through a variety of pathophysiological mechanisms and share common risk factors, such as genetic mutations, dyslipidemia, hyperuricemia, hyperglycemia, hypertension, insulin resistance, and obesity [16, 34]. LRP5 participates in lipid and glucose metabolism, and genetic polymorphism of LRP5 has been identified as contributing factor for metabolic disorders, which are determinants of cardiovascular disease and also closely associated with NAFLD [27, 35]. In this study, the relationship of the *LRP5* rs556442 polymorphism with the risks of NAFLD and CHD were explored in a Chinese Han population for the first time. The results of this study show that the distribution of *LRP5* rs556442 differed significantly between patients with only CHD and those with both NAFLD and CHD, and that the *LRP5* rs556442 GG genotype contributed to the risk of NAFLD in CHD patients. In addition, *LRP5* rs556442 AA + AG genotype correlated with the risk of obese NAFLD, but this association was not significant after adjustment for sex and age.

LRP5 is one of the LDL cholesterol receptors and is widely expressed in various tissues. LRP5 is known to participate in adipogenesis by down-regulating adipogenic transcription factors and also to regulate insulin secretion and cholesterol metabolism [25–27, 36]. In 2006, Guo et al. explored the effects of *LRP5* gene polymorphisms on obesity in a Caucasian population, and they found that intronic variants in the *LRP5* gene were significantly related to the risk for obesity [37]. In 2019, Adabi et al. conducted a cross-sectional study in Iranian postmenopausal women to explore the effect of the *LRP5* rs556442 polymorphism on basal metabolic rate and obesity. They found that among obese women, AG and AA genotype carriers had a lower basal metabolic rate than GG genotype carriers, and a lower basal metabolic rate will further aggravate obesity. Therefore, the AA/AG genotype was regarded as a risk factor for obesity in these patients [30]. In the present study, the *LRP5* rs556442 AA + AG genotype was associated with an increased BMI in all study participants and in obese NAFLD patients of the Chinese Han population, and these findings are consistent with the previous studies.

Prior research has shown that hepatic steatosis can contribute to the progression of CHD through increased inflammation in the local environment and endothelial dysfunction [38]. In the present study, CHD patients with the *LRP5* rs556442 GG genotype had an increased risk of developing NAFLD, which indicates that detection of the *LRP5* rs556442 GG genotype should be included in NAFLD screening efforts in CHD patients. No relationships between *LRP5* rs556442 and the risks of NAFLD, CHD, or the combination of NAFLD and CHD were found in this study. However, the pathogenesis of both NAFLD and CHD is complex and dependent on the combined actions of multiple factors. Therefore, single risk factors such as the *LRP5* rs556442 polymorphism may be masked by the influence of other factors. Although the present study did not find any significant associations between the *LRP5* rs556442 polymorphism and these diseases, the role of *LRP5* rs556442 in NAFLD, CHD, and NAFLD + CHD patients remains to be confirmed. In addition, the strong linkage disequilibrium between *LRP5* rs556442 and other polymorphisms may affect the effect of *LRP5* rs556442 on the risk of NAFLD, CHD, and NAFLD + CHD. Therefore, further studies should also explore these issues.

Serum AST is mainly produced in the liver and heart. When liver cells and cardiomyocytes are injured, AST in the cytoplasm can enter the blood, leading to an increase in the serum AST concentration. Therefore, a high serum AST level in clinical practice is usually related to liver cell or cardiomyocyte injury [39–41]. In this study, the *LRP5* rs556442 A allele carriers among NAFLD + CHD patients had elevated serum AST levels compared to the non-carriers, which suggests that *LRP5* rs556442 A allele carriers with NAFLD and CHD might be more prone to hepatocyte or cardiomyocyte damage. In addition, the levels of LDL in the CHD group and NAFLD + CHD group were inferior to that in the controls in present study, and this phenomenon might have been caused by the use of lipid-lowing drugs by patients with CHD and NAFLD + CHD.

The main strength of this study was the finding that the *LRP5* rs556442 GG genotype could increase the risk of NAFLD in CHD patients, which suggests that LRP5 rs556442 genotype can potentially be considered for screening purposes in the future if further research confirms these findings in other populations as well. The findings of this study provide insight for a new method to predict the risk of NAFLD in patients with CHD. This study also has several limitations. All patients with NAFLD were diagnosed by ultrasound rather than liver biopsy; therefore, diagnostic error may exist in this research. Also, the number of participants in this study was relatively small, which may affect the results. Finally, all of the participants were Chinese Han, and thus, the association of *LRP5* rs556442 with the risks of NAFLD, CHD, and the combination of NAFLD + CHD need to be verified in other ethnicities.

## Conclusions

This study explored the associations between the *LRP5* rs556442 polymorphism and the risks of NAFLD, CHD, and the combination of NAFLD + CHD in Chinese Han patients. The distributions of *LRP5* rs556442 genotypes differed between the CHD and NAFLD + CHD groups, and the *LRP5* rs556442 GG genotype increased the risk of NAFLD in CHD patients. In addition, the *LRP5* rs556442 AA + AG genotype was associated with an increased BMI in obese NAFLD patients, but this association was not statistically significant after adjustment for sex and age. Further studies are needed to verify the association of *LRP5* rs556442 polymorphism and the risks of NAFLD, CHD, and combined NAFLD + CHD in other countries and ethnicities, and the underlying mechanism by which *LRP5* rs556442 affecting the risk of NAFLD in patients with CHD needs to be clarified. Overall, the findings of this study provide insight for a new method to predict the risk of NAFLD in patients with CHD.

## Data Availability

All data generated or analyzed during this study are included in this published article.

## Abbreviations

ALP: Alkaline phosphatase
ALT: Alanine aminotransferase
AST: Aspartate aminotransferase
BMI: Body mass index
BMR: Basal metabolic rate
CHD: Coronary heart disease
CI: Confidence interval
CSC: Chinese Society of Cardiology
FPG: Fasting blood glucose
GGT: γ-Glutamyl transpeptidase
HDL: High density lipoprotein
LDL: Low density lipoprotein
LRP5: Low-density lipoprotein receptor-related protein 5
NAFL: Nonalcoholic simple fatty liver
NAFLD: Non-alcoholic fatty liver disease
NASH: Nonalcoholic steatohepatitis
OR: Odds ratio
SD: Standard deviation
TBIL: Total bilirubin
TC: Total cholesterol
TG: Triglycerides
